# Examining outcome of early physician specialist assessment in injured workers with shoulder complaints

**DOI:** 10.1186/s12891-015-0488-3

**Published:** 2015-02-18

**Authors:** Helen Razmjou, Dragana Boljanovic, Sandra Lincoln, Chris Geddes, Iona Macritchie, Caterina Virdo-Cristello, Robin R Richards

**Affiliations:** Holland Orthopedic & Arthritic Centre, Sunnybrook Health Sciences Centre, 43 Wellesley Street East, Toronto, ON M1Y 1H1 Canada; Department of Physical Therapy, Faculty of Medicine, University of Toronto, Toronto, Canada; Sunnybrook Research Institute, Sunnybrook Health Sciences Centre, Toronto, Canada; Division of Orthopedic Surgery, Department of Surgery, McMaster University, Hamilton, Canada; Division of Orthopedic Surgery, Department of Surgery, Sunnybrook Health Sciences Centre, Toronto, ON Canada; Department of Orthopedic Surgery, Faculty of Medicine, University of Toronto, Toronto, Canada

**Keywords:** Descriptive study, Early assessment, Injured workers

## Abstract

**Background:**

There is minimal research on demographics, type of injury and diagnosis of injured workers with shoulder problems. The purposes of this study were: 1) to document the demographics of patients with shoulder complaints referred to an Early Shoulder Physician Assessment (ESPA) Program and to describe the recommended management, and 2) to examine the relationship between patient characteristics and their subjective complaints of pain and functional difficulty.

**Methods:**

This study involved a retrospective review of electronic files of injured workers mostly seen within the first 16 weeks of injury or recurrence. Measures of functional difficulty and pain were the Quick Disabilities of the Arm, Shoulder and Hand (QuickDASH) and Numeric Pain Scale (NPS).

**Results:**

Files of 550 consecutive patients, 260 females (47%), 290 men (53%) were examined. The average age was 49 (SD = 11, range 22–77), with 28 (5%) patients being 65 years of age or older. Patients who were not working were the most disabled group based on Quick DASH (F = 49.93, p < 0.0001) and NPS (F = 10.24, p = 0.002). Patients who were working full time performing regular duties were the least disabled according to both measures, the QuickDASH (F = 10.24, p = 0.002) and NPS (F = 7.57, p = 0.006).

Patients waiting more than 16 weeks were slightly older (53 years of age vs. 49, p = 0.045) than those who met the criteria for early assessment with similar levels of pain and functional difficulty. Biceps pathology had the highest prevalence (37%). Full thickness tear had a prevalence of 14%. Instability, labral lesions and osteoarthritis of glenohumeral joint were uncommon conditions (3, 2 and 1% respectively). Fifty-five patients (10%) were surgical candidates and had higher scores on QuickDASH (F = 7.16, p = 0.008) and NPS (F = 4.24, p = 0.04) compared to those who did not require surgery.

**Conclusions:**

This study provides information on characteristics and prevalence of important variables in injured workers with shoulder problems and highlights the impact of these characteristics on pain and disability.

## Background

Musculoskeletal injuries occurring at work are a significant contributor to disability and health care costs in the working population with recurrent disabling symptoms being common in some cases [1,2]. Recent studies [3] indicate that percent of all emergency department visits for some work-related musculoskeletal injuries conditions has increased from 20% in 2004 to 23% in 2011. When frequency and cost of injury is taken into consideration, shoulder injuries are among the most expensive single injury types [4] and remain prevalent in the workplace environment [5-10]. These injuries have a significant negative effect on work productivity [11,12] and are often associated with poor surgical recovery [13-15]. Unfortunately, research studies focusing on shoulder injuries in adults have commonly been oversimplified in their representation of individuals injured in the workplace often categorizing injured workers based solely on their compensation status [16] rather than exploring type or severity of injury or reasons for failure to management. The classification of injured workers in this simplistic fashion may have contributed to inaccurate claims and perpetuated stigmas regarding the prognosis, treatment effect and overall outcomes of injured workers with disorders of the shoulder.

The limited literature suggests that patient’s demographics [17,18] and suboptimal education or work environment [19-21] are among important prognostic factors in patients with shoulder conditions. Apart from patient characteristics, timeframe and expertise of the clinicians appear to impact injured workers management [22]. An early assessment can reduce complications associated with chronic disability [23] and an expert evaluation can reduce the cost of unnecessary investigations sometimes ordered by primary health care providers [22,24]. In a study by Savoie et al. [22], use of an early referral system, as opposed to a traditional ‘gatekeeper’ approach (i.e. where care and potential referral to an orthopedic specialist is managed by the primary care physician), resulted in significant cost reductions (approximately $75 000 per patient). In addition to a 10-fold decrease in direct and indirect medical costs, patients in the ‘early referral’ group experienced significant decreases in time spent waiting for surgery (3.9 months in early referral versus 10.1 in ‘gatekeeper’ system) and time off work (6.6 versus 17.1 months).

As a part of improving care of injured workers in Canada, the Ontario Workplace Safety and Insurance Board (WSIB) initiated a new service specific to shoulder injuries beginning in November, 2012 [25]. This service involves an early expert assessment through Shoulder and Elbow Specialty Clinics for those workers who have not progressed in their recovery, have not returned to work (RTW) or progressed in their RTW plan within 16 weeks.

Personal and life style characteristics, the type of injury and occupational characteristics are important predictors of recovery [17,18,26-28]. Exploring the relationship between these important variables with pain and loss of function, may help to design better programs to facilitate the management and improve the outcome of injured workers with shoulder conditions.

The purposes of this study were: 1) to document the demographics of patients with shoulder complaints referred to an Early Shoulder Physician Assessment (ESPA) Program and to describe the recommended management, and 2) to examine the relationship between patient characteristics and their subjective complaints of pain and functional difficulty.

## Methods

This study involved a retrospective review of the electronic files of patients seen from the beginning of the program in November 2012 to July of 2014 at the Holland Orthopedic and Arthritic Centre, Toronto, Canada. Approval for using the existent data was obtained from the Research Ethics Board of the Sunnybrook Health Sciences Centre (project # 114–2014).

### Patient population

All of the patients had an accepted shoulder claim by the WSIB with a diagnosis of bursitis, impingement syndrome, rotator cuff tendinitis, sprain/strain, a partial or full thickness rotator cuff tear, instability, or superior labral lesions within 16 weeks of the injury or recurrence.

The assessment at the ESPA Program was conducted at the Holland Centre, which has a dedicated program for assessment and treatment of injured workers providing both conservative and surgical management. The ESPA Assessments are performed by an orthopedic surgeon and a physical therapist. The orthopedic surgeons have subspecialty in shoulder surgery and physical therapists have Masters’ degree or post graduate training and experience in this area. The assessments involve history taking and a clinical examination as well as arranging expedited investigations, conducting therapeutic cortisone injections and recommending treatment or surgery as needed. The information collected by examiners is entered into a fillable PDF form by the assessing physical therapist. The form includes details on the patient’s work status, the mechanism of injury, current symptoms, medication, investigations performed, relevant past medical history, life style factors, investigations performed, and physical examination. At the end of the assessment a clinical diagnosis is provided, and recommendations are made regarding further investigations, consultations and/or treatment along with appropriate worker accommodations and a return to work plan. Patients who may be candidates for surgery would then be referred to the Surgical Specialty Program, where they could undergo expedited surgery if required.

Patient oriented outcome measures completed at the time of assessment include the QuickDASH [29] and the Numeric Pain Scale (NPS). The QuickDASH is an upper extremity outcome measure with 11 questions and uses a Likert scale [29]. The NPS uses a 0 to 10 scale with 0 being no pain and 10 being worst imaginable pain. Higher numbers of QuickDASH and NPS indicate more pain and loss of function. Both measures have established acceptable validity and reliability in patients with shoulder complaints [18,29,30].

### Statistical analysis

Descriptive statistics [number (n), mean, standard deviation (SD), median, minimum, and maximum] were performed for demographics. Age was categorized into two groups: 1) working age group (<65 years of age) and 2) 65 and older. Number and percentage of patients who underwent diagnostic investigations or were referred to the Shoulder Specialty Program (SSP) and were booked for surgery were calculated. Subgroup analyses were conducted to examine potential differences in demographics and pain (as measured by NPS) and functional difficulty (as measured by QuickDASH) between patients a) who waited longer than 16 weeks versus those who did not, and b) patients who were surgical candidates vs. those who did not require surgery. In addition, the relationship between pain and functional difficulty and work status was examined. The Chi square (*χ*^2^) tests were used for categorical data and parametric and non-parametric analyses were used for continuous data as appropriate.

## Results

### Demographics

Five hundred and fifty consecutive patients were seen by an orthopedic surgeon and a physical therapist. Table 1 demonstrates the demographics of the sample studied. The percentage of men and women was similar with 260 females (47%) and 290 (53%) males. The average age was 49 (SD = 11), ranging from 22–77, with 28 (5%) patients being 65 years of age or older. Hypertension was the most common (97, 18%) reported associated comorbidity. Forty-seven percent of workers reported alcohol consumption and 30% were tobacco smokers. The most common mechanism of injury documented in the form involved lifting or pushing/pulling (40%). Falling on the outstretched hand was the least common injury (5%). The majority of patients (50%) were taking NSAID, with 44% taking a variety of analgesics including narcotics (Table 2).

**Table 1:** **Demographics of patients**

**Variables**	**Mean (SD) or N (%)**
**Timeframe (days) §**	79 (23), Range: 26–174
<16 weeks	521 (95%)
>16 weeks	29 (5%)
**Age (years)**	49 (11), Range: 22–77
<65 years	522 (95%)
65 and older	28 (5%)
**Sex**	
Male	290 (53%)
Female	260 (47%)
**Comorbidity**	
Hypertension	97 (18%)
Diabetes	42 (8%)
Cardiovascular disease	15 (3%)
Osteoporosis	6 (1%)
Recent infection	2 (<1%)
On Corticosteroids	3 (<1%)
**Life style**	
Alcohol consumption	259 (47%)
Smoker	170 (30%)
Inactive life style	115 (21%)
**Side of Shoulder Injury**	
Right	339 (62%)
Left	223 (41%)
12 patients had bilateral complaints	
**Hand dominance**	
Right	680 (91%)
Left	71 (9%)
**Mechanism of Injury**	
Lifting/pulling/pushing	219 (40%)
Repetitive activities/insidious	79 (14%)
Direct trauma	62 (11%)
Fall on point of shoulder	60 (11%)
Traction injury	39 (7%)
Fall on outstretched hand	27 (5%)
**Previous conditions**	
Previous work-related injury	143 (26%)
Previous non-work related injury	78 (14%)
Previous shoulder surgery	10 (2%)

§:Date of injury to date of assessment.

**Table 2:** **Disability and work status**

**Disability and pain rating**	
Numeric Pain rating	5.83 (2), Range: 0–10
QD (5 missing data)	56.45 (21), Range: 0–100
**Medication to date**	
NSAID	277 (50%)
Analgesics	244 (44%)
Muscle relaxants	34 (6%)
**Work status at the time of assessment**	
Regular job, regular hours (QD: 44/NPS: 5.76)	67 (12%)
Regular job, modified hours (QD: 52/NPS: 5.55)	11 (2%)
Modified job, regular hours (QD: 53/NPS: 5.16)	284 (52%)
Modified job, modified hours (QD: 62/NPS: 5.87)	62 (11%)
Not working (QD: 68/NPS: 6.40)	123 (22%)
**RTW recommendations**	
Pre-injury	30 (5%)
Pre-injury accommodated	426 (77%)
Alternate job	69 (13%)
No RTW	4 (<1%)
**Ability at the time of assessment**	
Full	16 (3%)
Required accommodation	531(97%)
**Status of functional abilities**	
Achieved full functional ability	3 (0.6%)
Full functional abilities expected	370 (67%)
Full functional abilities not expected	43 (8%)

QD: Quick-DASH (higher numbers indicate more disability).

NPS: Numeric Pain Score (higher numbers indicate more pain).

### Criteria for referral to EPSA

Although the inclusion criterion for referral to the ESPA program was 16 weeks or less since the time of injury, the wait time from the date of injury to the date of assessment varied from 26 to 174 days in our sample, with 29 patients (5%) waiting more than 16 weeks (group1) and 521 patients waiting ≤16 weeks (group 2). Patients who waited longer than 16 weeks (group 1) were slightly older (53 years of age vs. 49, p = 0.045) than group 2. There was no gender discrepancy between groups. Groups had comparable level of pain and disability as measured by the numeric pain scale (group1:5.93 vs group2: 5.83, p = 0.71) and Quick DASH (group1:51 vs group2: 57, p = 0.17). Management of these patients did not differ in terms of having more investigations, referral for surgical consultation or requiring surgery.

### Work status and disability

Twenty-six percent (143) of the patients had a previous related work injury with 14% (78) reporting a non-work related shoulder injury (Table 1). Table 2 shows the mean score of the outcome measures for each working category. Only three percent (16) had achieved full abilities at the time of the assessment with 97% (531) requiring accommodation at their jobs. Patients who were not working were the most disabled group based on QuickDASH (F = 49.93, p < 0.0001) and NPS (F = 10.24, p = 0.002). Patients who were working full time performing regular duties were the least disabled according to both measures: QuickDASH (F = 10.24, p = 0.002) and NPS (F = 7.57, p = 0.006).

### Investigations and diagnostic categories

Most patients referred to the ESPA program had at least one investigation ordered by their family physician with only 7% not having any investigations at all. Twenty three percent of patients were referred for further examination by the orthopedic surgeon in the ESPA program and 2% were awaiting investigations ordered by their family physician.

Table 3 shows the complete list of diagnoses made by the examiners. Prevalence of biceps pathologies was 202 (37%) in our study, of which 34% had an investigation that confirmed the biceps pathology (136 US and 53 MRI). A small proportion of the patients (3%) had a clinical diagnosis of biceps pathology based on tenderness, biceps deformity or a positive Yergason’s test. Full-thickness tears of the rotator cuff tendons had a prevalence of 14%. All Rotator cuff full thickness tears were diagnosed based on MRI or US performed prior to referral to the clinic.

**Table 3:** **Investigations and diagnosis**

**Investigations to date**	
None	41 (7%)
X-rays	346 (63%)
US	329 (60%)
MRI	126 (23%)
CT scan	7 (1%)
EMG	8 (1%)
**Further diagnostic testing**	138 (25%)
Diagnostic testing arranged by GP§	9 (2%)
Diagnostic testing ordered by ESPA program	129 (23%)
• MRI	82 (15%)
○ Not completed	4 (<1%)
• US	7 (1%)
• MRA	5 (1%)
• EMG	6 (1%)
• CT scan	2 (<1%)
**Diagnostic Categories**	
Biceps tendonitis/tear	202 (37%)
Bursitis	182 (33%)
RC partial thickness tear	119 (22%)
RC tendonitis	101 (18%)
RC full thickness tear	74 (14%)
Subacromial impingement	63 (11%)
Adhesive capsulitis	50 (9%)
AC joint OA or osteolysis	22 (4%)
Instability	14 (3%)
Labral lesions	9 (2%)
GH joint OA	7 (1%)
**Cervical spine**	
Abnormal clinical findings	164 (30%)
Abnormal neurological findings	50 (9%)
**Abnormal pain response**	43(8%)

§: Investigation arranged by GP but was not available at the time of assessment.

EMG: Electromyography, ESPA: Early Shoulder Physician Assessment, CT Scan: Computerized Tomography Scan, GH: Glenohumeral, GP: general Practitioner, MRA: Magnetic Resonance Arthrogram, MRI: Magnetic Resonance Imaging, US: Ultrasound.

No significant difference was found between men and women in the distribution of more minor rotator cuff pathology (tendonitis, bursitis, partial thickness tears) or biceps pathology (p > 0.05). However men had a statistically significant higher rate of full-thickness tears than women (57/290 = 20% vs. 18/260 = 7%, *x*^2^ 18.78, p < 0.0001).

Instability and labral lesions were not very common (3% and 2% respectively) in the sample studied. Similarly, osteoarthritis of the glenohumeral joint was an uncommon condition (1%) and was typically observed in association with other injury related conditions (e.g. impingement, rotator cuff tear, adhesive capsulitis). Cervical spine radiculopathy was common, involving 30% of the patients with 9% of the patients having neurological findings.

### Management

Table 4 displays treatment recommendations and a summary of barriers to recovery. Ninety-one percent of patients were encouraged to receive or continue with active treatment, with a much smaller group (18%) getting advice on medication. A small percentage (10%) received a therapeutic corticosteroid injection. Fifty-six patients (10%) were felt to have optimized medical management and were discharged.

**Table 4:** **Recommendations and barriers to recovery**

**Treatment Recommendations**	
Active treatment	503 (91%)
Medication	101(18%)
Referral to SSP clinic	77 (14%)
Surgery indicated	55 (10%)
Surgery booked	40 (7%)
Therapeutic injection	57 (10%)
No further medical treatment	56 (10%)
Referral to function and pain specialty clinic	3 (0.6%)
**Barriers to RTW**	
Workers factors	88 (16%)
Injury related medical factors	38 (7%)
Psychological factors	18 (3%)
Pre-existing medical factors	17 (3%)
Work place factors	28 (5%)

SSP: Specialty Surgical Program.

Seventy-seven patients (14%) were deemed to require further assessment and potentially surgery and were referred to the Surgical Specialty Program (SSP). Fifty-five patients (71% of the referred group and 10% of the entire sample) were deemed surgical candidates and 40 (7%) patients were booked for surgery within the time frame of the manuscript preparation. Patients who were referred to the SSP were more disabled (based on the QuickDASH collected on initial assessment at the ESPA clinic) than those who were not referred (mean 61 vs. 56, F = 4.69, p = 0.03). Similarly, surgical candidates had higher QuickDASH (mean 65 vs.56, F = 7.16, p = 0.008) and pain (6.27 vs. 4.80, F = 4.24, p = 0.04) than those who did not require surgery.

## Discussion

This study presents the demographics of a large consecutive sample of injured workers who underwent an early assessment through a newly established program. In addition, we explored the relationship between perceived pain and ability to function and important patient-related factors.

### Work status

In our sample, QuickDASH was affected by reporting psychological issues (mean of 75 vs. 56 respectively, p = 0.005). The QuickDASH was also correlated with work status with the score being lowest in workers doing regular duties. However, non-working individuals were not necessary experiencing more psychological problems. Pichora and Grant who investigated the difference between working and non-working individuals with upper extremity injuries found that non-workers suffered from poorer mental functioning [31]. Overall, facilitating a safe return to work will help to avoid long term or permanent disability and reinforcing the availability of modified duties, shorter hours and a more supportive work environment should not be underestimated in injured workers with physically demanding jobs.

### Investigations

There are clinical practice guidelines for ordering costly investigations such as MRI for workers with acute occupational low back injuries [32] but we are not aware of such guidelines for shoulder injuries. The American College of Radiology (ACR) provides rating for routinely used investigations for shoulder pain [33] and shoulder trauma [34] but these guidelines may not be completely applicable to injuries occurring in the workplace environment. Presently, clinicians dealing with injured workers order investigations based on the mechanism of injury, physical examination findings and patient characteristics. Future research should focus on providing guidelines on the type of investigations that should be ordered for specific occupational injuries. Having access to an investigation algorithm will assist family physicians and other primary care providers to order appropriate investigations without adding unnecessary cost to the system.

### Diagnostic categories

The injured workers in our sample had a variety of diagnoses, mostly related to bursitis, tendonitis and partial-thickness tears of the rotator cuff and biceps which is consistent with common pathologies of the shoulder joint. Full-thickness tears comprised a small percentage of the diagnostic categories (14%) with the biceps pathologies making up the highest proportion of clinical diagnosis (37%). Prevalence of biceps pathology in injured workers is not clearly defined in the literature. The overall prevalence of full-thickness tears in non-work related injuries in patients seen in secondary clinics is reported at about 8% [35]. However, the prevalence of tears depends on the age of the sample included and status of injury (work-related vs. non-work-related). More specifically, in the 50 year old age group of the general population, the prevalence of rotator cuff tears is reported at 11%, increasing to 15% in the 60 year olds, to 27% in 70 year olds, and to 37% in 80 year olds [36]. Krishnan et al. [37] who investigated patients younger than 40 years of age, reported a much higher prevalence of 43%. Dwyer et al. [38] found that 29% (24/84) of patients younger than 55 years of age had a shoulder work-related injury as compared to 11% (28/260) in older patients.

Adhesive capsulitis had a rate of 9% in injured workers seen in this early assessment program. Rate of adhesive capsulitis has been reported to be at approximately 2% in the general population, increasing drastically in diabetic patients [39,40]. We are not aware of the incidence of this condition in injured workers and further study of this is warranted as this condition is associated with significant disability.

Instability related pathologies and labral tears had the lowest prevalence in our sample varying from 2 to 3%. In a large epidemiology of surgical interventions of upper extremity, Jain et al. found a prevalence of 13% for instability and SLAP repairs [41]. The prevalence of SLAP repair in injured workers has been reported to be at 25% [42]. Our low prevalence is potentially due to a low number of MR-arthrogram studies that are preferred in the diagnosis of labral pathology. The clinical significance of labral pathology in the absence of instability is not fully understood.

As seen in our sample, cervical spine radiculopathy appears to be a common associated comorbidity which may be missed in the busy clinics of primary family physicians. Having expert examiners is expected to help identifying associated pathologies more accurately that contribute to patients symptoms.

The gender distribution was of interest with men and women being fairly similar when all pathologies were considered together. However, consistent with the literature [38,43-46], men had a higher incidence of rotator cuff tears than women, potentially due to a higher rate of trauma or involvement in labor intensive occupations [47].

### Management

Management recommendations for injured workers included active treatment physiotherapy programs (91%), medication (18%) including analgesic and non-steroidal antiinflammatories, referral for surgery (14%), and corticosteroid injections (10%) including subacromial and intraarticular glenohumeral. Less than 1% of the patients were referred to a pain clinic for a multidisciplinary approach. Abnormal pain response which is clinically associated with poorer outcome had a low prevalence of 8%. Our results may indicate that an early referral to shoulder specialty clinics is associated with less exaggerated pain behaviors and more importantly helps with promoting a more active treatment and less reliance on chronic pain management.

In the sample studied, 10% of the injured workers did not require any medical management and 10% were surgical candidates. Efficiency improvements are possible in early assessment models of care if patients who are likely to require surgical treatment (i.e. full-thickness rotator cuff tears) were referred directly to the clinics with access to surgical resources. Access to a management algorithm may facilitate the referrals made by case managers or family physicians.

Establishing a timely recommendation for appropriate surgical intervention may help to avoid problems associated with delayed treatment (namely weakness, stiffness and pain) and could lead to improved outcomes and faster recovery. In our sample, 14% of patients assessed in the ESPA clinic required a referral to SSP. This result shows the importance of early assessment for an early diagnosis followed by a direct path through to treatment and recovery. Early assessment of patients with rotator cuff pathology has shown to significantly decrease medical costs and time spent waiting for surgery and being off work [22].

Access to programs for early assessment, diagnosis and management of shoulder conditions are still not that common and more improvement could be made in this area. Improving the timely access to appropriate management is expected to reduce the costs it places on the health care system and workforce. Future studies may provide evidence for a reduction in costs and improvement in expenditure in this area.

### Limitations

This descriptive study involved a retrospective analysis of data of consecutive injured workers. Although the data were extracted from standardized forms, the analysis was limited to available variables. For example, mental health, alcohol consumption and smoking were limited to categorical data of yes and no. In addition, we had access only to the total score of the QuickDASH. This measure is reported to show factorial structure inconsistency in patients with shoulder conditions mostly related to one of the questions that explores neurological symptom of tingling [48,49]. Future studies should address the potential shortcomings of this measure in patients with work-related shoulder condition.

## Conclusions

This study provides descriptive information on a large sample of injured workers seen within a short period of time following injury. Early diagnosis made by expert clinicians with appropriate investigations is expected to facilitate more timely conservative or surgical management, leading to better overall management.

## Abbreviations

ACR: American college of radiology
ESPA: Early shoulder physician assessment
Quick DASH: Quick disabilities of the arm, shoulder and hand
US: Ultrasound
MRI: Magnetic resonance imaging
NPS: Numeric pain scale
NSAID: Non-steroidal anti-inflammatory drugs
RTW: Return to work
SD: Standard deviation
SSP: Specialty Surgical Program
WSIB: Workplace safety and insurance board
